# Serum 25-hydroxyvitamin D concentration is associated with device-estimated sleep metrics in healthy young and early middle-aged adults

**DOI:** 10.1093/sleepadvances/zpaf077

**Published:** 2025-10-29

**Authors:** Michele N D’Agata, Elissa K Hoopes, Thomas Keiser, Freda Patterson, Benjamin C Brewer, Melissa A Witman

**Affiliations:** Department of Kinesiology and Applied Physiology, College of Health Sciences, University of Delaware, Newark, DE, United States; Department of Health Behavior and Nutrition Sciences, College of Health Sciences, University of Delaware, Newark, DE, United States; Department of Health Behavior and Nutrition Sciences, College of Health Sciences, University of Delaware, Newark, DE, United States; Department of Health Behavior and Nutrition Sciences, College of Health Sciences, University of Delaware, Newark, DE, United States; Center for Biostatistics and Health Data Science, College of Science, Virginia Polytechnic Institute and State University, Roanoke, VA, United States; Department of Kinesiology and Applied Physiology, College of Health Sciences, University of Delaware, Newark, DE, United States

**Keywords:** vitamin D, sleep, actigraphy, melatonin, young adults

## Abstract

**Study Objectives:**

We tested associations between serum 25-hydroxyvitamin D concentration ([25(OH)D]) and device-estimated sleep metrics, including sleep duration, sleep efficiency, sleep duration regularity, sleep timing regularity, and sleep regularity index (SRI), in young and early middle-aged adults (18–45 years). We also assessed the mediating effect of nighttime melatonin (urinary 6-sulfatoxymelatonin (aMT6s) excretion) on these associations.

**Methods:**

Participants (*n* = 79) completed 14 days of wrist actigraphy. Fasted blood sampling was performed to quantify serum [25(OH)D]. First morning void was used to quantify overnight urinary aMT6s excretion, normalized to creatinine clearance. Associations between [25(OH)D] and sleep metrics were evaluated using linear regression (model 1). Separate models adjusted for age, sex, race, and body fat % (model 2), season of testing, caffeine consumption, and education level (model 3), and device-estimated moderate-to-vigorous physical activity (model 4; *n* = 68).

**Results:**

Serum [25(OH)D] was positively associated with sleep duration, sleep efficiency, and SRI, and negatively associated with sleep duration regularity, sleep onset timing regularity, and sleep midpoint timing regularity in model 1 (all *p* < .03) and model 4 (all *p* < .02). In model 2, serum [25(OH)D] remained significantly associated with sleep duration only (*p* = .036). In model 3, serum [25(OH)D] remained significantly associated with all sleep metrics (*p* < .02) except sleep duration regularity and SRI. Serum [25(OH)D] was not associated with aMT6s:creatinine, indicating no grounds for performing mediation analyses.

**Conclusions:**

Serum [25(OH)D] is independently associated with several sleep metrics in healthy adults. However, nighttime melatonin concentration did not mediate these associations, thus other mechanistic pathways must be considered.

## Teaser

**Statement of Significance** Here, we utilize 14 days of wrist actigraphy to evaluate cross-sectional associations between serum 25-hydroxyvitamin D ([25(OH)D]) concentration and device-estimated sleep metrics in a sample of healthy adults without evidence of clinical sleep disorders. Our results highlight significant associations between [25(OH)D] and several sleep metrics in this population, extending findings previously observed in only older adults and individuals with sleep disorders. We also newly assess the influence of urinary aMT6s excretion, an indicator of nighttime melatonin production, on the association between [25(OH)D] and sleep, given that melatonin has been proposed as a potential mechanism linking the two. Findings show that nighttime melatonin does not mediate the associations between [25(OH)D] and device-estimated sleep metrics, suggesting other mechanistic pathways should be explored.

## Introduction

Vitamin D insufficiency and deficiency are highly prevalent, impacting an estimated 25% and 41% of U.S. adults, respectively [1]. Vitamin D is primarily obtained from direct cutaneous ultraviolet B radiation, although it can also be obtained through consumption in foods or supplements [2]. Common barriers to achieving sufficient vitamin D status include increased time spent indoors, use of sunscreen and protective clothing, seasonal, regional, and daytime variation in the zenith angle of the sun, increased skin pigmentation, obesity, and age [3]. Current evidence implicates 25-hydroxyvitamin D ([25(OH)D]) concentration, the readily measured metabolite of vitamin D in the blood, in the regulation of sleep based largely on observational studies demonstrating that low serum vitamin D is correlated with poor quality sleep and short sleep duration [4]. Thus, low circulating [25(OH)D] may have major public health relevance given that poor sleep is associated with reductions in cognition, mental health, and physical health, including cardiovascular disease (CVD), which is the leading cause of death in U.S. adults [5, 6].

Adults are recommended to achieve ≥7 h of sleep per night, although more than 1 in 4 Americans report not meeting sleep duration recommendations [7, 8]. Other dimensions of sleep are also recognized as important components of overall sleep health, including sleep efficiency, sleep continuity, day-to-day regularity in sleep duration and timing, and sleep quality [9]. Large observational studies employing actigraphy-derived estimates of sleep report that [25(OH)D] is positively associated with sleep duration in a nationally representative sample of older adults [10], and is positively associated with sleep duration, sleep efficiency, and sleep continuity in a community sample of older men [11]. Moreover, patients with sleep disorders such as narcolepsy with cataplexy, obstructive sleep apnea, and insomnia have been shown to have significantly lower [25(OH)D] and a higher prevalence of vitamin D deficiency as compared with individuals without sleep disorders [12–14]. Findings to date suggest a beneficial role of vitamin D on sleep health in older adults and individuals with sleep disorders, although data evaluating associations between [25(OH)D] and device estimates of sleep in otherwise healthy young adults free of sleep disorders are lacking. Additionally, no studies have evaluated the association between [25(OH)D] and sleep regularity index (SRI), which is a novel and important metric of sleep health strongly associated with CVD risk, as well as circadian timing among college students [15–17].

Melatonin is a prominent hormone in the regulation of sleep, which is synthesized in response to low-light conditions to help synchronize the sleep–wake cycle to the light–dark cycle within the ~24-h day [18]. In addition to facilitating sleep timing and regularity, melatonin increases sleep propensity to help maintain continuous sleep throughout the night [18]. Mechanistically, vitamin D is proposed to influence sleep through the melatonin pathway by increasing the activity of tryptophan hydroxylase 2, the enzyme responsible for regulating the conversion of tryptophan into 5-hydroxytryptophan, which is further metabolized to produce melatonin [4, 19]. Vitamin D receptors have also been detected within several other regions of the brainstem known to have roles in sleep regulation, including the hypothalamus, prefrontal cortex, and substantia nigra [4]. However, the potential mediating effect of nighttime melatonin production on the association between [25(OH)D] and sleep health has not been explored.

Thus, the purpose of this study was to investigate cross-sectional associations between serum [25(OH)D] and actigraphy-derived estimates of sleep health, including sleep duration, sleep efficiency, sleep duration regularity, sleep timing regularity, and SRI, in apparently healthy young and early middle-aged adults. The secondary aim was to examine the association between serum [25(OH)D] and overnight urinary 6-sulfatoxymelatonin (aMT6s) excretion, a well-established proxy of circulating plasma melatonin concentration [20, 21], and to explore if aMT6s concentration mediated the association between [25(OH)D] and actigraphy-derived sleep metrics. We hypothesized that increased serum [25(OH)D] would be associated with increased sleep duration, higher sleep efficiency, more regular sleep duration as assessed by sleep duration SD, more regular sleep timing as assessed by sleep onset SD and sleep midpoint SD, a higher SRI, and higher aMT6s concentration. We further hypothesized that aMT6s concentration will at least partly mediate associations between [25(OH)D] and device-estimated sleep metrics.

## Materials and Methods

### Study participants and protocol

Data used in this study are from two separate protocols within the same research laboratory approved by the Institutional Review Board at the University of Delaware (IRB Study No. 1704969 and 1957713). Protocols were identical in the methods of serum [25(OH)D] and aMT6s quantification, as well as for collection of device-estimated sleep metrics, as written below. Data from IRB Study No. 1957713 includes baseline data from participants from a larger clinical trial that is registered on ClinicalTrials.gov (ID: NCT05656742). Each study was conducted in accordance with the ethical standards of the *Declaration of Helsinki* and written informed consent was obtained from all participants. Apparently healthy adults between the ages of 18 and 45 years were recruited from the University of Delaware and the surrounding Newark, DE region. Participants included cisgender men and women who were generally healthy, non-shift working, without evidence of clinical sleep disorders or conditions known to affect sleep (e.g. depression), not taking medication or supplements known to affect sleep, non-smoking, had a body mass index (BMI) between 18.5 and 35 kg/m^2^, and had screening resting systolic blood pressure <140 and diastolic blood pressure <90 mmHg. Participants were also excluded, if they had a history of any major chronic diseases or conditions, including cardiovascular, renal, metabolic, autoimmune, or cancerous conditions, as well as a recent history of coronavirus disease 2019 infection (<60 days) or vaccination (<14 days). All female participants were premenopausal, not pregnant, and not currently breastfeeding.

After providing study informed consent, screening procedures required participants to complete a review of medical history and medication use and additional screening for the presence of insomnia (Insomnia Severity Index score ≥ 15) [22] and sleep apnea (STOP-bang score ≥ 3) [23]. Seated resting blood pressure was assessed in triplicate (Omron 5 Series, BP7200). Height and weight were measured for the calculation of BMI and body fat percentage was estimated via bioelectrical impedance analysis (Tanita TBF-300A, Arlington Heights, IL). Eligible participants were equipped with an accelerometer (Actiwatch Spectrum Plus; Philips-Respironics, Inc.) that they were instructed to wear on their non-dominant wrist for 14 consecutive days and nights for estimation of habitual sleep metrics. On the final night of sleep monitoring and immediately prior to returning to the lab (day 15), participants completed an overnight urine collection (first morning void). Participants were instructed to report to the laboratory on day 15 during the morning hours (between 07:00 and 11:30 am.), following an overnight fast, without caffeine for ≥12 h, without alcohol or exercise for ≥24, without over-the-counter medications or anti-inflammatory drugs for ≥72 h prior to the visit, and to avoid taking any vitamins or supplements on the morning of the visit. Upon arrival at the laboratory, intravenous blood sampling was performed during the morning hours for later assessment of serum [25(OH)D] as well as for the clinical assessment of fasting blood glucose and a lipid panel (Study No. 1704969: Quest Diagnostics, Inc., Philadelphia, PA; Study No. 1957713: LabCorp Testing Services).

### Quantification of serum [25(OH)D]

A fasted venous blood sample was collected during the morning hours (between 07:00 and 11:30 am.) and serum was aliquoted and stored at −80°C for later analysis of [25(OH)D] using a 25-OH vitamin D ELISA Assay Kit according to manufacturer specifications (Eagle Biosciences, Amherst, NH, USA). Samples were assayed in triplicate; intra-assay coefficients of variation (CV) were <6.9% and inter-assay CVs were <8.6%. Clinically, serum [25(OH)D] concentrations ≥30 ng/mL are considered sufficient, concentrations <30 ng/mL but ≥20 ng/mL are considered insufficient, and concentrations <20 ng/mL are considered vitamin D deficiency [24].

### Device-estimated sleep metrics

Sleep metrics were estimated via wrist actigraphy (Actiwatch Spectrum Plus) as previously described [25]. In short, participants were instructed to wear the Actiwatch continuously on their non-dominant wrist for 14 days and nights, in conjunction with a standardized daily sleep diary to assist with sleep–wake scoring [26]. To estimate habitual sleep health metrics, a conservative minimum of 10 days and nights of wear was required for inclusion in analyses [27]. Data were collected in 30-s epochs and processed by trained investigators (M.N.D., E.K.H., and T.K.) using both participants’ standardized sleep diaries and Philips Actiware software (version 6.1.0). Nights with >1 h of missing data were considered invalid [28]. Rest intervals were identified using a standardized protocol that incorporates sleep diaries, activity levels, and “lights out” [28, 29]. Sleep–wake scoring was based on the medium threshold setting for sleep/wake detection using the algorithm provided by the manufacturer. Metrics of sleep duration (total time scored as sleep between sleep onset and sleep offset) and sleep efficiency (total sleep time divided by total time in bed, expressed as a percentage) were calculated for each night of wear and mean values were generated to characterize habitual sleep duration and sleep efficiency for each participant over the 14-day wear period. Sleep duration regularity was calculated as the SD of nightly sleep duration (sleep duration SD). Sleep timing was operationalized as the timing of sleep onset (clock time at start of each nocturnal sleep period) and the timing of sleep midpoint (clock time halfway between sleep onset and sleep offset). Sleep timing regularity was calculated as the SD of sleep onset timing (sleep onset SD) and the SD of sleep midpoint timing (sleep midpoint SD). Sleep regularity index was statistically computed as the likelihood that any two timepoints 24 h apart were the same sleep/wake state across all days [30]. To complement device estimates of sleep, participants also completed the Pittsburgh Sleep Quality Index (PSQI), a valid and reliable subjective assessment of sleep quality over the last month [31], within 1 week of completing actigraphy.

### Quantification of overnight urinary aMT6s excretion normalized to urinary creatinine clearance

On the morning after the final night of sleep monitoring (day 15) participants were instructed to complete an at-home urine collection for the assessment of overnight aMT6s excretion normalized to urinary creatinine clearance. Participants were instructed to discard their last urinary void of the evening, right before bedtime, and then collect all voids that occur between sleep onset and sleep offset, as well as their first void after awakening, prior to returning to the lab that same morning. Urine was mixed by inversion and 1 mL aliquots were frozen at −80°C for later analysis of aMT6s concentration (IBL International GmbH, Melatonin-Sulfate EIA, Hamburg, Germany), normalized to urinary creatinine concentration (Quidel Corporation, MicroVue Creatinine EIA, San Diego, CA, USA). Both aMT6s and creatinine were quantified via ELISA kits according to manufacturer specifications. All samples were assayed in duplicate. aMT6s intra-assay CV was <12.2% and inter-assay CV was <14.9%. Creatinine intra-assay CV was <2.1% and inter-assay CV was <6.9%. Urinary aMT6s:creatinine ratio (ng/mg) was used for all analyses to represent nighttime urinary aMT6s excretion normalized to creatinine. Values were obtained in *n* = 77 participants due to *n* = 2 participants having aMT6s concentrations out of detectable range.

### Other measures

Participants who completed IRB Study No. 1704969 also completed device-estimates of physical activity (*n* = 68), concurrent with the 14-day sleep monitoring period. Physical activity was estimated via waist-worn accelerometry (ActiGraph wGT3X+, ActiGraph, LLC) as described [25]. Participants were instructed to wear the ActiGraph during all waking hours for 14 consecutive days that overlapped with sleep monitoring. Data were collected in 10-s epochs and processed using ActiLife software (version 6.13.4). Wear time was first validated using the Troiano algorithm [32], and periods of non-wear were removed from analyses. Activity intensity was estimated using activity counts per minute (CPM) according to the Freedson Combination 1998 algorithm, with moderate-vigorous physical activity (MVPA) quantified as >1951 CPM [33]. A minimum of 10 h of wear time per day was considered valid [34]. Minutes spent in MVPA was derived for each day and average daily MVPA was calculated for each participant over the 14-day wear period.

### Covariates

Age (in years), sex, participant race/ethnicity (self-report), and body fat % were obtained at screening. Data were also coded based on season of testing at the start of sleep monitoring (spring, summer, fall, and winter), which was included as a covariate. Additional covariates included number of beverages containing caffeine (report in number of drinks per week) and education level (reported using the PCS3 Socioeconomic Status Questionnaire, Carnegie Mellon University). For the subset of participants with physical activity data, average daily MVPA was included as covariates. All covariates were selected on a theoretical basis, as each of these variables have been shown to have an established association with either serum [25(OH)D] or sleep outcomes [35–40].

### Statistical analyses

Descriptive statistics, including mean and SD for continuous variables and count (*n*) and percentage for categorical variables, were used to describe our sample. Separate linear regression models for each sleep metric were generated to ascertain the association with serum [25(OH)D]. The first step of the model included serum [25(OH)D] (model 1). For the second step, age, sex, race, and body fat % were added (model 2). In the third step, age, sex, race, and body fat % were removed and season of testing, habitual caffeine consumption, and education level were added (model 3). The fourth step only included serum [25(OH)D] and daily MVPA, which was obtained in a subset of participants (*n* = 68) (model 4). Multicollinearity was assessed for all covariates and no associations were found between independent variables in each model. The standard mediation model as proposed by Baron and Kenny [41] in 1986 would be used to assess the mediating effect of aMT6s normalized to creatinine concentration on the association between serum [25(OH)D] and each actigraphy-derived sleep metric if model assumptions were met. Specifically, to prove mediation, the predictor (serum [25(OH)D]) must be significantly associated with the hypothesized mediator (aMT6s:creatinine), and the magnitude of the predictor’s effect on the outcome (sleep metrics) must significantly decrease when controlling for the hypothesized mediator. Above analyses were performed with *jamovi* (Version 2.3.26) [Computer Software], Sydney, Australia. R statistical software was used to compute SRI, which was determined as the likelihood that any two timepoints (epoch-by-epoch) 24 h apart were the same sleep/wake state, across all days using the formula and methods from Windred et al. [30]. Individuals will only display sleep patterns that range between an SRI of 0 (random) and ±100 (periodic), however, values <0 are theoretically possible (e.g. sleep for 24 h, wake for 24 h, etc.), but unlikely to be observed.

Statistical significance was set a priori at α ≤ 0.05; after correcting for multiple testing via the Benjamini–Hochberg procedure, the new per-model significance threshold was α^*^ ≤ 0.0375. Standardized beta (β) values reported in Table 3 indicate model effect sizes where effect sizes between 0.10 and 0.29 are small, 0.30 and 0.49 are medium, and 0.50 or greater are large. Post hoc power analyses were performed to determine the statistical power for associations between vitamin D and sleep metrics (G^*^Power). In-text linear regression results are presented as unstandardized B values and 95% confidence intervals (CIs). Summary data are presented as mean ± SD or *n* (%).

## Results

**Figure 1:**
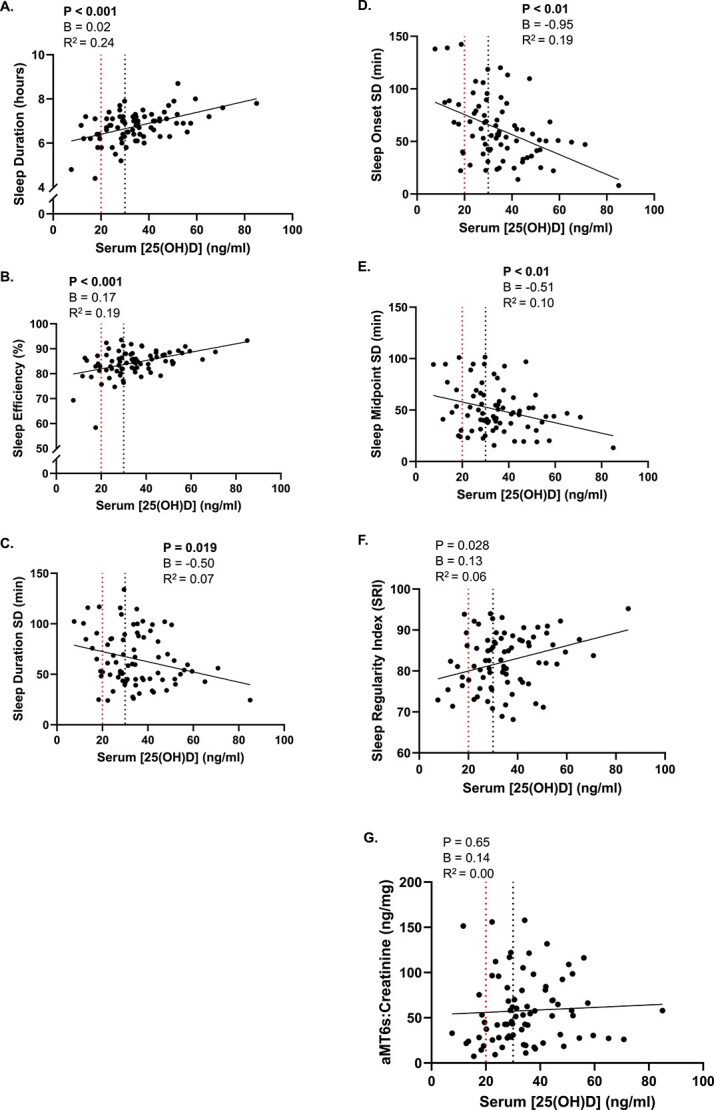
Unadjusted associations between serum [25(OH)D] and device-estimated sleep metrics. Scatterplots of sleep duration (A), sleep efficiency (B), sleep duration SD (C), sleep onset SD (D), sleep midpoint SD (E), sleep regularity index (F), and ^#^urinary aMT6s:Creatinine ratio (G) with serum [25(OH)D]. The vertical dashed line at 20ng/ml represents the clinical cut-off value between vitamin D insufficiency and deficiency (<20 ng/mL) and the vertical dashed line at 30 ng/ml represents the clinical cut-off value between vitamin D sufficiency and insufficiency (<30 ng/mL). Linear regression lines represent unadjusted models. B, unstandardized coefficient; SD, standard deviation. Significance was set at α ≤ 0.0375, *p*-values in **bold** indicate significance. ^#^Urinary aMT6s:Creatinine ratio quantified in *n* = 77 participants.

Participant characteristics are presented in Table 1. Participants (*n* = 79) included generally healthy young adults (66% female, 53% non-Hispanic White) that were well-educated (80% obtained a bachelor’s degree or higher). Device-estimated sleep metrics and self-report sleep metrics are presented for all participants in Table 2. Scatterplots are displayed in Figure 1 to demonstrate unadjusted associations between serum [25(OH)D] and device-estimated sleep metrics. Serum [25(OH)D] was positively associated with sleep duration (*p* < .001, Figure 1A), sleep efficiency (*p* < .001, Figure 1B), and SRI (*p* = .028, Figure 1F), and negatively associated with sleep duration SD (*p* = .02, Figure 1C), sleep onset SD (*p* < .001, Figure 1D), and sleep midpoint SD (*p* < .01, Figure 1E); however, no association was observed with urinary aMT6s:creatinine ratio (*p* = .65, Figure 1G). Serum [25(OH)D] was also not associated with global PSQI score (unstandardized coefficient (B) = 0.00 score, 95% CI = −0.03 to 0.03, *p* = .81) or self-report sleep duration (B = 0.01 h, 95% CI = −0.00 to 0.03, *p* = .07). Post hoc power analyses revealed the statistical power for the independent associations between serum [25(OH)D] and each sleep metric at our given sample size and reported effect sizes as follows: sleep duration (99.81%), sleep efficiency (98.25%), sleep duration SD (62%), sleep onset SD (98.58%), sleep midpoint SD (78.57%), and SRI (74.49%). Table 3 demonstrates that serum [25(OH)D] remained significantly associated with sleep duration only after multivariable adjustment in model 2, sleep duration, sleep efficiency, sleep onset SD, and sleep midpoint SD after multivariable adjustment in model 3, and all sleep metrics in model 4.

**Table 1:** Participant characteristics and sleep metrics (*n* = 79)

	Mean ± SD or *n* (%)
Age, years	29 ± 7
Sex	
Female	52 (66)
Male	27 (34)
Race/ethnicity	
White, non-Hispanic	42 (53)
Black, non-Hispanic	16 (20)
Hispanic	6 (8)
Asian	12 (15)
Other	3 (4)
Body mass index, kg/m^2^	24.1 ± 3.0
Systolic/diastolic blood pressure, mmHg	115 ± 9/71 ± 7
Caffeine consumption, drinks/week	7 ± 7
Fasting clinical blood markers	
Blood glucose (mg/dL)	89 ± 7
Total Cholesterol (mg/dL)	169 ± 28
LDL Cholesterol (mg/dL)	93 ± 24
HDL Cholesterol (mg/dL)	61 ± 14
Triglycerides (mg/dL)	74 ± 32
Serum [25(OH)D], ng/mL	34.5 ± 13.9
Vitamin D sufficient (≥30.0 ng/mL)	45 (57)
Vitamin D insufficient (20.0-29.9 ng/mL)	23 (29)
Vitamin D deficient (< 20.0 ng/mL)	11 (14)
Vitamin D supplement use, yes	13 (16)
Season of testing	
Spring	21 (27)
Summer	25 (32)
Fall	13 (16)
Winter	20 (25)
Education	
Didn’t finish high school	0 (0)
High school graduate or GED	5 (6)
Less than 2 years of college	4 (5)
2 years of college or more	7 (9)
College graduate (4+ year program)	27 (34)
Master’s degree (or postgraduate training)	28 (36)
Doctoral degree	8 (10)

Values are presented as means ± SD or *n* (%).

**Table 2:** Sleep-related metrics (*n* = 79)

Device-estimated sleep metrics	Mean ± SD
Nights of sleep	13 ± 1
Sleep duration, hours	6.8 ± 0.7
Sleep efficiency, %	84 ± 5
Sleep duration SD, minutes	65 ± 27
Sleep onset SD, minutes	61 ± 30
Sleep midpoint SD, minutes	50 ± 23
Sleep regularity index	82.2 ± 7.2
^#^aMT6s:Creatinine ratio, ng/mg	57.9 ± 37.2
Self-report sleep assessment	
PSQI, global score	4 ± 2
PSQI, sleep duration, hours	7.4 ± 0.8
^#^Average daily MVPA, minutes	55 ± 29

Values are presented as means ± SD or *n* (%). ^#^Values quantified in a subset of participants: a6MTs:creatinine ratio, *n* = 77; average daily MVPA, *n* = 68; MVPA, moderate-to-vigorous physical activity; PSQI, Pittsburgh Sleep Quality Index; SD, standard deviation.

**Table 3:** Adjusted associations between serum [25(OH)D] and device-estimated sleep metrics: sleep duration (A), sleep efficiency (B), sleep duration SD (C), sleep onset SD (D), sleep midpoint SD (E), and sleep regularity index (F)

A. Sleep duration, hours			95% Confidence interval			
Predictor	B	SE	Lower	Upper	β	*P*-value
*Model 1*						
Serum [25(OH)D], ng/mL	0.02	0.01	0.01	0.03	0.49	**<.001**
*Model 2*						
Serum [25(OH)D], ng/mL	0.01	0.01	0.00	0.03	0.26	**.036**
*Model 3*						
Serum [25(OH)D], ng/mL	0.03	0.01	0.02	0.04	0.53	**<.001**
*Model 4*						
Serum [25(OH)D], ng/mL	0.03	0.01	0.01	0.04	0.50	**<.001**
**B. Sleep efficiency, %**			**95% Confidence interval**			
**Predictor**	**B**	**SE**	**Lower**	**Upper**	**β**	***P*-value**
*Model 1*						
Serum [25(OH)D], ng/mL	0.17	0.04	0.09	0.25	0.43	**<.001**
*Model 2*						
Serum [25(OH)D], ng/mL	0.09	0.05	- 0.01	0.19	0.24	.067
*Model 3*						
Serum [25(OH)D], ng/mL	0.18	0.04	0.09	0.27	0.47	**<.001**
^#^ *Model 4*						
Serum [25(OH)D], ng/mL	0.12	0.04	0.05	0.20	0.36	**.002**
**C. Sleep duration SD, minutes**			**95% Confidence interval**			
**Predictor**	**B**	**SE**	**Lower**	**Upper**	**β**	***P*-value**
*Model 1*						
Serum [25(OH)D], ng/mL	−0.50	0.21	−0.92	−0.09	−0.26	**.019**
*Model 2*						
Serum [25(OH)D], ng/mL	−0.36	0.27	−0.90	0.18	−0.19	.188
*Model 3*						
Serum [25(OH)D], ng/mL	−0.01	0.00	−0.01	0.00	−0.23	.065
^#^ *Model 4*						
Serum [25(OH)D], ng/mL	−0.59	0.24	−1.07	−0.11	−0.29	**.016**
**D. Sleep onset SD, minutes**			**95% Confidence interval**			
**Predictor**	**B**	**SE**	**Lower**	**Upper**	**β**	***P*-value**
*Model 1*						
Serum [25(OH)D], ng/mL	−0.95	0.22	−1.40	−0.50	−0.44	**<.001**
*Model 2*						
Serum [25(OH)D], ng/mL	−0.49	0.27	−1.04	0.06	−0.22	.081
*Model 3*						
Serum [25(OH)D], ng/mL	−0.91	0.23	−1.37	−0.45	−0.42	**<.001**
^#^ *Model 4*						
Serum [25(OH)D], ng/mL	−1.13	0.25	−1.63	−0.63	−0.49	**<.001**
**E. Sleep midpoint SD, minutes**			**95% Confidence interval**			
**Predictor**	**B**	**SE**	**Lower**	**Upper**	**β**	***P*-value**
*Model 1*						
Serum [25(OH)D], ng/mL	−0.51	0.18	−0.86	−0.15	−0.31	**.006**
*Model 2*						
Serum [25(OH)D], ng/mL	−0.07	0.21	−0.50	0.35	−0.04	.736
*Model 3*						
Serum [25(OH)D], ng/mL	−0.50	0.19	−0.88	−0.11	−0.31	**.012**
^#^ *Model 4*						
Serum [25(OH)D], ng/mL	−0.68	0.20	−1.09	−0.28	−0.39	**.001**
**F. Sleep regularity index**			**95% Confidence interval**			
**Predictor**	**B**	**SE**	**Lower**	**Upper**	**β**	***P*-value**
*Model 1*						
Serum [25(OH)D], ng/mL	0.13	0.06	0.01	0.24	0.25	**.028**
*Model 2*						
Serum [25(OH)D], ng/mL	−0.02	0.07	−1.15	−0.12	−0.03	.812
*Model 3*						
Serum [25(OH)D], ng/mL	0.11	0.06	−0.02	0.23	0.21	.086
^#^ *Model 4*						
Serum [25(OH)D], ng/mL	0.18	0.07	0.05	0.31	0.32	**.009**

Model 1, unadjusted; model 2 adjusts for age, sex, race, and body fat %; model 3 adjusts for season of testing, habitual caffeine consumption, and education level; model 4 adjusts for average daily moderate-to-vigorous physical activity (MVPA). ^#^Model 4 was tested in a subset of participants (*n* = 68). B, unstandardized coefficient; SE, standard error of unstandardized coefficient; β, standardized coefficient; SD, standard deviation. Significance was set at α ≤ 0.0375, *p*-values in **bold** indicate significance.

Serum [25(OH)D] was not associated with aMT6s:creatinine (Figure 1G) and urinary aMT6s:creatinine ratio was not associated with any device-estimate sleep metrics (*p* > .31 for all, data not shown). Based on these null associations, we did not meet the assumptions for conducting mediation analyses and thus conclude no mediating effect of urinary aMT6s:creatinine ratio on any of the associations between serum [25(OH)D] and actigraphy-derived sleep metrics.

## Discussion

Findings from this study indicate that serum [25(OH)D] was significantly associated with all sleep metrics in the absence of covariates (model 1). Specifically, in unadjusted analyses, every 10 ng/mL increase in serum [25(OH)D] was significantly associated with a 0.20 h (12 min) increase in sleep duration, a 1.7% increase in sleep efficiency, a 5 min decrease in sleep duration SD, a 9.5 min decrease in sleep onset SD, a 5.1 min decrease in sleep midpoint SD, and a 1.3% increase in SRI. Further, serum [25(OH)D] remained significantly associated with sleep duration after multivariable adjustment including age, sex, race, caffeine consumption, season of testing, and physical activity among others in our sample of generally healthy young and early middle-aged adults. However, there appears to be some influence from covariates on the association between the other sleep metrics and serum [25(OH)D] as statistical significant was lost following adjustment in some models. To our knowledge, this is the first study to test these associations in a young, generally healthy population of adults without evidence of clinical sleep disorders. This study also newly demonstrates that nighttime melatonin production was not associated with serum [25(OH)D], and thus, in our dataset, is not a mediator of the associations between serum [25(OH)D] and device-estimated sleep metrics, suggesting that other mechanisms may be responsible for the associations observed between these variables.

Our study is in agreement with findings from large population-based cohorts conducted largely in middle-aged and older adults, including the National Health and Nutrition Examination Survey (NHANES) and the Multi-Ethnic Study of Atherosclerosis (MESA), which have reported independent associations between serum [25(OH)D] and sleep [10, 13]. Specifically, in the NHANES cohort (2005–2006), serum [25(OH)D] was found to be inversely associated with self-reported daytime sleepiness and presence of insomnia after controlling for several covariates, including age, sex, race/ethnicity, BMI, education, marital status, family income, self-report physical activity, cigarette smoking, alcohol use, caffeine intake, and anti-depressant use [13]. In the MESA cohort (2000–2002) increased serum [25(OH)D] was found to be significantly associated with increased actigraphy-derived sleep duration obtained over 5–12 nights, as well as increased time in rapid eye movement sleep as assessed by polysomnography after adjustment for several variables, including age, sex, race/ethnicity, waist circumference, education, family income, physical activity, smoking status, alcohol use, and anti-depressant use [10]. Other studies evaluating associations between [25(OH)D] and actigraphy-derived estimates of sleep have also been conducted in older adult men [11], night-shift versus day-shift workers [42], and adults with and without depression [43], with each of these studies reporting a positive association between [25(OH)D] and actigraphy-derived sleep duration. Moreover, [25(OH)D] was also found to be significantly and positively associated with actigraphy-derived sleep efficiency in older adult men [11]. In the present study, we extend these findings to a sample of generally healthy young and early middle-aged adults. Elucidating a potential relationship between serum [25(OH)D] and sleep health is noteworthy given that poor sleep health early in life has been linked to increased risk of high blood pressure, obesity, depression, and anxiety, which are known to contribute to the development of CVD [44–46].

Importantly, the several dimensions of sleep health investigated in the present study, including short sleep duration, low sleep efficiency, irregular sleep duration, irregular sleep timing, and SRI have all been independently linked to adverse cardiovascular health outcomes [9, 15]. Actigraphy-derived short sleep duration has been associated with higher atherosclerotic burden and incidence of hypertension among middle-aged and older adults [47, 48]. Lower objectively estimated sleep efficiency from polysomnography has also been longitudinally associated with increased risk of incident CVD in a community sample of older adults [6], and decreased actigraphy-derived sleep efficiency has been associated with other strong predictors of CVD risk including decreased systolic blood pressure dipping and increased nocturnal heart rate in young adults, independent of sleep duration [49]. Finally, composite sleep regularity (SRI), irregular sleep duration, and sleep timing have each been reported to be associated with risk of cardiovascular events, independent of sleep duration and other traditional CVD risk factors [15, 50]. Notably, our laboratory has previously reported associations between sleep duration regularity and sleep onset timing regularity and circulating immune cells [51], as well as sleep duration regularity and lower-limb microvascular function [52] in young adults, suggesting that the adverse cardiovascular effects of irregular sleep are evident as early as young adulthood. The associations that we observed between serum [25(OH)D] and each of these dimensions of sleep may indicate a beneficial role for vitamin D in sleep health, however given the cross-sectional and observational nature of this study, it remains to be determined if vitamin D causally or indirectly influences sleep.

Still, studies providing a mechanistic link between serum [25(OH)D] and sleep are lacking. Nonetheless, evidence for a connection between serum [25(OH)D] and sleep is supported by double-blind randomized placebo-controlled vitamin D supplementation interventions. For example, in a sample of otherwise healthy young adults with vitamin D deficiency, a single dose of 100 000 IU of vitamin D3 (cholecalciferol) significantly increased serum [25(OH)D] and significantly decreased daytime fatigue as assessed by the Fatigue Assessment Scale after 4 weeks as compared with a placebo group [53]. Other double-blind randomized placebo-controlled trials report similar findings among young and middle-aged adults with sleep disorders [54] as well as in middle-aged adults with insomnia [55]. Specifically, bi-weekly supplementation with 50 000 IU of vitamin D versus placebo for 8 weeks significantly improved (decreased) global PSQI score and significantly increased self-report sleep duration in young and middle-aged adults with sleep disorders (determined via the Petersburg’s Sleep Index) as compared with a placebo group [54]. In patients with insomnia, daily supplementation with 1500 IU of vitamin D versus placebo for 10 weeks significantly improved insomnia symptoms as assessed by the Insomnia Severity Index [55]. However, in the present study we did not detect a cross-sectional association between serum [25(OH)D] and subjective sleep quality or self-report sleep duration as assessed by the PSQI, which may be population- and study design-specific as it is likely that larger effects could be observed in adults with sleep disorders following an acute period of vitamin D supplementation. Still, a limitation of previous work evaluating the effect of vitamin D supplementation on sleep is the reliance on self-report rather than objective sleep estimates, of which the latter may provide a more accurate representation of sleep health [56, 57], and thus remains an important area of investigation in future interventions. In the present study, only 13 participants reported taking supplements containing vitamin D, although the exact dosage and frequency of supplementation was not recorded. As expected, the participants that reported taking vitamin D supplements had higher serum [25(OH)D] compared with those who did not report taking vitamin D supplements (48.5 ng/mL versus 31.7 ng/mL, *p* < .01); however, aMT6s concentration was not statistically different between groups (47.3 ng/mg versus 59.9 ng/mg, *p* = .28). Further, no statistical differences were observed among sleep metrics between groups (data not shown). However, given the small sample size of participants taking supplements, the potential for healthy user bias among those taking supplements, and the lack of information regarding dosage, these findings should be interpreted with caution and require further investigation.

No study to date has attempted to interrogate potential mechanisms linking serum [25(OH)D] and sleep health. Thus, in the present study, we newly assessed a potential mediating effect of nighttime melatonin production on the association between [25(OH)D] and device-estimated sleep metrics. On a cellular level, vitamin D has been implicated in both the homeostatic regulation and the circadian regulation of sleep. Specifically, vitamin D has been shown to increase activity of the enzyme tryptophan hydroxylase 2 (TPH2), which aids in the downstream production of melatonin [19]. Through genome sequencing TPH2 has been confirmed to contain two vitamin D response element sequences that are associated with transcriptional activation of the gene [58]. Further, treatment of human brain glioblastoma/astrocytoma (U87 MG) cells with 1,25(OH)D, the active metabolite of vitamin D, was found to significantly increase TPH2 mRNA in a dose-dependent manner [19]. The effect of vitamin D on the circadian process of sleep has also been demonstrated through treatment of adipose-derived stem cells with 1,25(OH)D, which elicited synchronization of *BMAL1* and *Per2* gene expression, demonstrating a profile similar to adipose-derived stem cells synchronized by a serum shock [59]. In contrast to this mechanistic work, our study did not detect an association between serum [25(OH)D] and melatonin production, or a mediating effect of nighttime melatonin production as measured by urinary aMT6s:creatinine on the association between serum [25(OH)D] and device-estimated sleep metrics. These null findings may be due to methodological limitations. For example, we only evaluated a single night of urinary aMT6s excretion which may not be reflective of habitual urinary aMT6s excretion, and thus, may have weakened potential associations. Regardless, previous studies demonstrate that quantification of creatinine-adjusted urinary aMT6s excretion using a single morning void has a strong correlation with total and peak nocturnal plasma melatonin concentrations in middle-aged and older women [60], has good sensitivity and specificity in identifying nocturnal plasma melatonin concentrations in healthy young men [61], and has good reproducibility among premenopausal women [62]. Instead, the circadian process of sleep and/or other internal factors, external factors, and behaviors known to influence sleep health may have masked any mediating effect of aMT6s on the association between [25(OH)D] and device-estimated sleep metrics [63]. It is also possible that the effect of melatonin on device estimates of sleep is relatively small and was therefore unable to be detected with our cross-sectional study design and with our current sample size.

### Experimental considerations

This study has several strengths. First, vitamin D was found to be independently associated with actigraphy-derived sleep duration, sleep efficiency, and sleep onset SD in the presence of several potential confounding variables. This study is also strengthened by the inclusion of multiple assessments of sleep health including device-estimated sleep duration, sleep efficiency, and sleep duration regularity, sleep timing regularity, a composite SRI, subjective sleep quality, and first morning void urinary aMT6s excretion. We also employed a robust 14 days of wrist actigraphy to estimate sleep health metrics, allowing us to uniquely test metrics of sleep regularity in this context while using a highly reproducible sleep watch that has been validated against polysomnography in healthy adults [29]. Finally, although our sample size is relatively small compared to other population-based investigations, a notable strength of our study is the strict inclusion and exclusion criteria, where factors like location, age, BMI, medication use, smoking status, sleep disorders, and other comorbidities were all tightly controlled. This is in contrast to large epidemiological studies that often include several study sites, a large age range (e.g. 20–85 years), and participants with a variety of health conditions that are likely to affect sleep, such as depression, sleep disorders, type 2 diabetes, CVDs, and cancers.

This study also presents with limitations. Both serum [25(OH)D] and sleep health may be confounded by daily sun exposure, which was not directly assessed in this analysis, although we did adjust for season of testing in model 3, which had no impact on the association between [25(OH)D] and sleep duration, sleep efficiency, or sleep timing regularity. In this way, daytime sun exposure could also be a mechanism linking serum [25(OH)D] and sleep health as the sun’s light–dark cycle significantly influences the human sleep–wake cycle down to the genomic level [64]. However, information regarding light exposure obtained through windows, the use of sunscreen, or protective clothing while spending time outside (which all block UBV rays limiting vitamin D production) could alter the association between daily sun exposure and serum [25(OH)D], making it challenging to discern the mechanistic link with sleep. Similarly, exposure to artificial light at night can suppress nocturnal melatonin production [65]. Given the observational nature of the study, participants were not instructed to limit at-home light exposure on the night of urine sampling or throughout their participation. Furthermore, no information was collected regarding 24-h light exposure, a factor which the authors suggest should be recorded or considered in future work. There are also more comprehensive, sensitive, and robust methods for assessing nighttime or 24-h melatonin that should be considered in future work, including salivary dim-light melatonin onset for determining circadian phase timing [66] or 24-h urine collection where aMT6s is assayed from each void [67]. Additionally, findings from the present study would be strengthened by subsequent investigation of the effect of vitamin D supplementation on device estimates of sleep.

### Conclusions

In conclusion, we newly demonstrate that serum [25(OH)D] is associated with multiple device-estimated sleep health metrics in apparently healthy young adults, extending findings that have previously been observed in older adults and clinical populations. We also found that nighttime melatonin did not mediate the association between [25(OH)D] and device-estimated sleep metrics. Future work should evaluate the effect of vitamin D supplementation on device estimated sleep health as well as explore other potential mechanisms/biomarkers beyond melatonin that have the potential to link vitamin D with various dimensions of sleep health.

## Data Availability

The data that support the findings of this study are available from the corresponding author upon reasonable request.

## Clinical trial registration

Effects of Vitamin D on Cardiovascular Health in Black Women, https://clinicaltrials.gov/study/NCT05656742?locStr=Delaware&country=United%20States&state=Delaware&cond=Vitamin%20cD%20Deficiency&term=blood%20pressure&intr=Vitamin%20Dc&rank=1 Registered on ClinicalTrials.gov ID NCT05656742.
